# Complement C3a activates astrocytes to promote medulloblastoma progression through TNF-α

**DOI:** 10.1186/s12974-022-02516-9

**Published:** 2022-06-20

**Authors:** Biao Gong, Duancheng Guo, Chaonan Zheng, Zhen Ma, Jie Zhang, Yanghui Qu, Xinhua Li, Gen Li, Li Zhang, Yuan Wang

**Affiliations:** Laboratory of Molecular Neuropathology, Pediatric Cancer Center, College of Pharmaceutical Sciences, Soochow University, Suzhou, China

**Keywords:** Medulloblastoma, Astrocytes, C3a, TNF-α

## Abstract

**Background:**

Medulloblastoma (MB) is the most common malignant brain tumor in children. Approximately one-third of MB patients remain incurable. Understanding the molecular mechanism of MB tumorigenesis is, therefore, critical for developing specific and effective treatment strategies. Our previous work demonstrated that astrocytes constitute the tumor microenvironment (TME) of MB and play an indispensable role in MB progression. However, the underlying mechanisms by which astrocytes are regulated and activated to promote MB remain elusive.

**Methods:**

By taking advantage of *Math1-Cre/Ptch1*^*loxp/loxp*^ mice, which spontaneously develop MB, primary MB cells and astrocytes were isolated and then subjected to administration and coculture in vitro. Immunohistochemistry was utilized to determine the presence of C3a in MB sections. MB cell proliferation was evaluated by immunofluorescent staining. GFAP and cytokine expression levels in C3a-stimulated astrocytes were assessed by immunofluorescent staining, western blotting, q-PCR and ELISA. C3a receptor and TNF-α receptor expression was determined by PCR and immunofluorescent staining. p38 MAPK pathway activation was detected by western blotting. Transplanted MB mice were treated with a C3a receptor antagonist or TNF-α receptor antagonist to investigate their role in MB progression in vivo.

**Results:**

We found that complement C3a, a fragment released from intact complement C3 following complement activation, was enriched in both human and murine MB tumor tissue, and its receptor was highly expressed on tumor-associated astrocytes (TAAs). We demonstrated that C3a activated astrocytes and promoted MB cell proliferation via the p38 MAPK pathway. Moreover, we discovered that C3a upregulated the production of proinflammatory cytokines, such as IL-6 and TNF-α in astrocytes. Application of the conditioned medium of C3a-stimulated astrocytes promoted MB cell proliferation, which was abolished by preincubation with a TNF-α receptor antagonist, indicating a TNF-α-dependent event. Indeed, we further demonstrated that administration of a selective C3a receptor or TNF-α receptor antagonist to mice subcutaneously transplanted with MB suppressed tumor progression in vivo.

**Conclusions:**

C3a was released during MB development. C3a triggered astrocyte activation and TNF-α production via the p38 pathway, which promoted MB cell proliferation. Our findings revealed the novel role of C3a-mediated TNF-α production by astrocytes in MB progression. These findings imply that targeting C3a and TNF-α may represent a potential novel therapeutic approach for human MB.

**Supplementary Information:**

The online version contains supplementary material available at 10.1186/s12974-022-02516-9.

## Introduction

Medulloblastoma (MB), the most common malignant brain tumor in children, accounts for approximately 30% of brain tumors in children and has high mortality and poor prognosis [1, 2]. MB is classified into four main molecular subgroups: Wnt, Hedgehog (Hh), Group 3 and Group 4 [3]; among them, Hh-type MB, which is driven by aberrant activation of Hh signaling, represents more than 30% of the incidence [4]. MB is predominantly located in the cerebellum but spreads to other parts of the central nervous system, so aggressive multimodality standard therapies, including surgery, radiotherapy and chemotherapy, always cause severe side effects in patients, such as cognitive disorders, endocrine dysfunction and ataxia [5, 6]. Although some targeted therapies improve patient outcomes at the beginning of treatment, drug resistance soon arises due to treatment-induced gene mutations in tumor cells [7]. Therefore, multiangle/multitargeting therapeutic strategies urgently need to be developed. However, the mechanisms of MB tumorigenesis have not yet been completely elucidated, limiting the development of new strategies. Current hot spots of MB research focus on investigating the molecular profiles and mechanisms of tumor cells themselves [8]. In fact, nontumor cell populations in the tumor microenvironment (TME) of MB also deserve attention, since the TME plays essential roles in the tumorigenesis of many cancers and presents potential therapeutic targets [9].

Astrocytes are the most abundant glial cells in mammalian brains and express specific markers, such as S100β [10], astrocyte cell surface antigen 2 (ACSA-2) [11] and glial fibrillary acid protein (GFAP) [12–14]. Astrocytes support the development and function of neurons under physiological conditions [15, 16]. Published studies reported that astrocytes enhance tumor cell survival during chemotherapy [17], and astrocytes produce proinflammatory cytokines to promote glioma growth and metastasis [18–20]. These reports claimed that astrocytes are TME components in both primary and metastatic brain tumors. Consistent with these findings, our recent studies demonstrated for the first time that TAAs are the pivotal components in the MB TME, and play an indispensable role in not only primary but also relapsed MB progression [21, 22]. In these studies, we found that TAAs in MB upregulate numbers of gene expression as well as the ability to promote MB cell proliferation compared with primary astrocytes derived from normal mice. However, the mechanisms by which TAAs are regulated and activated to support MB have not been revealed. In current study, we aimed to investigate the underlying mechanisms by which astrocytes are modulated to construct the TME during MB development. Regarding astrocyte activation, Liddelow et al. summarized that astrocytes become reactive in the pathogenesis of neuroinflammation and ischemia [23]. Astrocytes were also shown to be activated in other neural dysfunctions, such as neural injury and neurodegenerative diseases [24–26]. Some research has reported that astrocyte activation in physiology and pathology is attributed to complement C3a. For instance, C3a stimulates astrocytes to produce growth factors to support neurons [27], and C3a promotes astrocyte survival in ischemia [28]. Moreover, C3a upregulates proinflammatory cytokine and chemokine expression in astrocytes [29, 30]. Here, we wondered whether C3a contributes to TAA activation during MB tumorigenesis.

Complement C3a is the product of complement system activation. The complement system, the critical component of the innate immunity, constitutes the first line of body defense. The complement system is composed of more than 30 proteins and can be activated through 3 different pathways: the classical, alternative, and lectin pathways. All 3 pathways merge at complement 3 (C3). Accompanied by complement activation, the C3 protein is cleaved to release the peptide C3a [31, 32]. C3a, also termed anaphylatoxin, is identified as a proinflammatory peptide. Through binding to its receptor (C3a receptor, C3aR), C3a triggers the migration and activation of inflammatory cells, including granulocytes, macrophages, mast cells and T cells etc. [33–37]. As a result, over activated C3a–C3aR signaling causes multiple diseases more than inflammation [38]. C3a–C3aR signaling has also been found to play a promotive role in cancers, including ovarian cancer, lung cancer and melanoma [39, 40]. In view of C3a’s functions in astrocyte activation and cancer regulation, combined with the fact that astrocytes are the major component of the TME of MB, we hypothesized that C3a may activate astrocytes and promote MB development.

## Materials and methods

### Animals

Wild-type (WT) *C57BL/6* mice and *CB17/SCID* mice were obtained from Beijing Vital River Laboratory Animal Technology Co., Ltd. *Math1-Cre/Ptch1*^*loxp/loxp*^ mice and *eGFP* mice were bred in our laboratory. *Math1-Cre/Ptch1*^*loxp/loxp*^ mice were generated by Dr. Yang as described previously [41]. In these mice, *Ptch1* is conditionally deleted in Math1-positive cells, which are MB original cells, granule neuron precursors (GNPs). These mice develop MB spontaneously starting from 6 to 8 weeks. Homozygous *Math1-Cre/Ptch1*^*loxp/loxp*^ mice were screened from heterozygous breeding pairs for the MB tumor model, and *Math1-Cre/Ptch1*^*wt/wt*^ littermates were used as controls. *Math1-Cre/Ptch1*^*loxp/loxp*^ mice were cross-mated with *eGFP* mice to obtain eGFP-positive MB cells. All mice were housed in a specific pathogen-free facility at School of Pharmaceutical Sciences, Soochow University.

### Primary cell isolation and culture

As described previously [21], for primary MB cell isolation and culture, tumor-bearing *Math1-Cre/Ptch1*^*loxp/loxp*^ or *Math1-Cre/Ptch1*^*loxp/loxp*^ /*eGFP* mice were sacrificed, and their heads were exposed. Tumor tissues were removed from the cerebella and digested in papain buffer consisting of 10 U/mL papain (Worthington), 250 U/mL DNase (Sigma) and 200 µg/mL l-cysteine (Sigma) for 30 min at 37 °C to acquire a single-cell suspension. These cells were used to generate a subcutaneous transplantation MB model. To obtain purified MB cells, the single-cell suspension was then separated through 35–65% Percoll medium (Sigma). MB cells were located in the 65% concentration layer. Tumor cells were suspended in NB-B27 culture medium (Neurobasal, 1 mM sodium pyruvate, 2 mM l-glutamine, 2% B27 supplement, all from Gibco, and 1% penicillin/streptomycin, Beyotime) and seeded on poly-d-lysine-coated coverslips placed in 24-well plates.

To isolate tumor-associated astrocytes (TAAs), a single-cell suspension of MB tissue was obtained as above. The cells were stained with APC-conjugated anti-ACSA-2 antibody (1:20, Miltenyi Biotec), and then TAAs were sorted and collected by FACS (Aria II, BD Bioscience) according to APC positive staining. For primary astrocyte culture, the cerebella of WT mice at postnatal day 2 (P2) were dissected and digested as described above to acquire a single-cell suspension, and cells were cultured in DMEM/F12 medium (HyClone) containing 10% fetal bovine serum (FBS, Gibco) and 1% penicillin/streptomycin. When the cells grew to 80% confluence, they were digested with 0.25% trypsin–EDTA (Beyotime) for passage. Cells were generally passed to two to three generations for experiments.

### Chemical and protein reagents

Recombinant mouse C3a, TNF-α and IL-6 were purchased from Novoprotein. In addition, recombinant mouse C3a from R&D Systems was used in some of the experiments as indicated. C3a was used for primary astrocyte stimulation at the indicated concentrations; TNF-α and IL-6 were administered to MB cells at lower (200 ng/mL) and higher (500 ng/mL) concentrations, and the data shown in the results are representative of the 500 ng/mL treatment groups. C3aR antagonist (SB290157), p38 inhibitor (SB203580) and TNF-α receptor antagonist (R-7050) were all purchased from ApexBio. SB290157 and SB203580 were used at 2 μM for astrocyte administration, and R-7050 was used at 500 nM for MB cell administration in vitro.

### Coculture of astrocytes and MB cells

Primary astrocytes and purified MB cells were mixed at a 1:10 ratio and cocultured in MB medium NB-B27 for 48 h. In certain experiments, astrocytes were pretreated with C3a or both C3a and p38 inhibitor for 24 h, and then the cells were harvested and washed with PBS for 3 times before being cocultured with MB cells. To collect astrocyte-conditioned medium, astrocytes were cultured in DMEM/F12 medium with C3a or vehicle as a control for 24 h, and the supernatant was removed before the addition of fresh medium. Astrocytes were cultured for another 24 h for conditioned medium collection. MB cells were then cultured in astrocyte-conditioned medium mixed with NB-B27 at a 1:1 ratio for 48 h.

### Subcutaneous transplantation MB model and treatment experiments

Four-to-six-week-old male *CB17/SCID* mice were subcutaneously injected with 2 × 10^6^ MB cells per mouse in the hind flanks. Tumor volume was measured and calculated as (*W*^2^ × *L*)/2 (*W*, width; *L*, length). When the tumor volume reached 200 mm^3^, the mice were randomly grouped and received treatments. For both in vivo treatment experiments, the C3aR antagonist SB290157 and the TNF-αR antagonist R-7050 were dissolved in 3% DMSO in MCT (0.5% methyl cellulose containing 0.2% Tween-80, Solarbio). MB tumor-bearing mice were injected intraperitoneally with SB290157 (30 mg/kg body weight), R-7050 (30 mg/kg body weight) or vehicle control once a day for 8 days at designated sites. Tumor volume was measured daily. Tumors were harvested at the end of treatment and underwent sectioning and RNA or protein sample preparation.

### Immunohistochemistry and immunofluorescent staining

Human MB paraffin sections were provided by Dr. Liu from Sanbo Brain Hospital Capital University and obtained from Children's Hospital of Soochow University, respectively. The use of the sections was reviewed and approved by the Ethics Committee of Soochow University. For immunohistochemistry, MB-bearing and WT normal mice were perfused, and cerebella and subcutaneous allografts were dissected and fixed in 4% paraformaldehyde (PFA, Macklin) for 24 h at 4 °C, dehydrated in 30% sucrose and embedded in paraffin, followed by sectioning at 6 μm thickness. MB paraffin sections were sent to Wuhan Servicebio Technology for immunohistochemistry staining of C3 and C3a. The antibodies used for detection were anti-human/mouse C3 polyclonal antibody (1:100, GeneTex), anti-human C3a monoclonal antibody, clone 2991 (1:200, Bio-Rad), anti-mouse C3a monoclonal antibody, and clone I87-1162 (1:200, BD Biosciences). Hematoxylin–eosin (H&E) staining was performed to compare the histological properties between WT normal cerebellum, primary MB and subcutaneous tumors following the standard protocol.

For immunofluorescent staining, tissues were harvested as above, frozen in Tissue Tek-OCT (Thermo Fisher Scientific), and then sectioned at 10–12 μm thickness. Sections were blocked with PBS containing 0.2% Triton X-100 (Biosharp) and 1% bovine serum albumin (BSA, Fisher bioreagents) for 1 h at room temperature. Then, the sections were incubated with primary antibody overnight at 4 °C. The next day, sections were exposed to secondary antibody conjugated with the appropriate fluorescein for 2 h at room temperature. DAPI (Beyotime) staining was performed for 10 min. Immunofluorescent staining for cells followed the same protocol. Before staining, cells were fixed in 4% PFA for 15 min and permeabilized with PBS containing 0.2% Triton X-100 for 10 min at room temperature. Finally, slides were mounted with Fluoromount-G (Southern Biotechnology) and visualized by a Nikon microscope. All antibodies were diluted in PBS containing 0.2% Triton X-100 and 1% BSA. The primary antibodies and dilution ratios were as follows: anti-C3aR (1:200, Bioss, 2955R), anti-Iba-1 (1:300, Invitrogen, MA5-27726), anti-Ki67 (1:200, Abcam, ab15580), anti-cleaved caspase-3 (1:200, Cell Signaling Technology, CST9661), and anti-GFAP (1:200, BD, 556330). The secondary antibodies and dilution ratios were as follows: goat anti-rabbit-Alexa Fluor 594 (1:200, Proteintech, SA00006-4), goat anti-mouse-Alexa Fluor 488 (1:200, Proteintech, SA00006-2), and goat anti-rabbit-Alexa Fluor 488 (1:200, Proteintech, SA00006-1).

### Western blotting

Cell lysate or MB tissue homogenate was prepared in RIPA buffer (Thermo Fisher Scientific) containing protease and phosphatase inhibitor cocktail (Roche), and the total protein concentration was evaluated using a BCA Protein Assay Kit (Beyotime). Then, equal amounts of protein samples were loaded and separated by 10% SDS–PAGE (Beyotime) and transferred onto PVDF membranes (Millipore). The membranes were blocked with TBS containing 0.05% Tween-20 and 5% defatted milk powder and incubated overnight at 4 °C with primary antibodies against GFAP (1:200, BD Biosciences, 556330), GAPDH (1:5000, Proteintech, 60004), total p38 (1:1000, Cell Signaling Technology, CST, 9212), phosphorylated p38 (1:1000, CST, 9215), β-Tubulin (1:10,000, Proteintech, 66240), phosphorylated Erk1/2 (1:1000, CST, 4370) and total Erk (1:1000, CST, 4695). The following day, the membranes were incubated with the HRP (horseradish peroxidase)-conjugated secondary antibodies anti-mouse IgG (1:5000, CST, 7076) and anti-rabbit IgG (1:5000, CST, 7074) at room temperature for 2 h. Bands were visualized using high-signal ECL substrate (Beyotime) and exposed on a ChemiScope 3300 mini (Clinx).

### ELISA (enzyme-linked immunosorbent assay)

Tissue homogenate and cultured cell conditioned medium were centrifuged, and the supernatant was collected to perform ELISA. The total protein concentrations of the samples were detected with a BCA Protein Assay Kit (Beyotime) and adjusted to be equal in each group. To detect C3a, a 96-well plate was precoated with purified anti-mouse C3a monoclonal antibody (1:500, clone I87-1162, BD Biosciences, 558250) overnight at 4 °C. The plate was washed with PBST (PBS containing 0.05% Tween-20) and blocked with 1% BSA in PBST. Samples and standards (purified mouse C3a protein, BD Biosciences, 558618) were added to the wells and incubated for 1 h at room temperature. Biotinylated anti-mouse C3a antibody (1:500, clone I87-419, 558251) was later used to bind to C3a antigen for 1 h at room temperature. Then, streptavidin–HRP (1:1000, BD Biosciences, 554066) was added and incubated for 30 min. Finally, the substrate TMB (Beyotime) was added for color development, and the reaction was stopped by 1 M H_2_SO_4_ 10 min later. The optical density (OD) in each well was read with a microplate reader at 450 nm. The C3a level was calculated according to the standard curve. TNF-α was determined utilizing a mouse TNF-α ELISA Kit (Fcmacs) in accordance with the manufacturer’s instructions.

### Conventional PCR and real-time quantitative PCR (qPCR)

Total RNA was extracted from cell lysate or tissue homogenate using TRIzol reagent (Sigma-Aldrich) in RNase-free conditions, and the purity of RNA was detected with a Nanodrop 2000 spectrophotometer (Thermo). cDNA was synthesized using oligo (dT) and Superscript II reverse transcriptase (Invitrogen). Conventional PCR was performed following a standard protocol, and PCR products were visualized in a 1.5% agarose gel. Quantitative PCRs were performed in triplicate using SYBR qPCR Master Mix (Vazyme Biotech) and the ABI 7500 TaqMan Real-Time PCR Detection System. The differences in mRNA expression were calculated by the 2^−ΔΔCt^ method. The primers (all for mouse species) used in the experiments included: *GAPDH* (forward: 5-CATCACTGCCACCCAGAAGACTG-3; reverse: 5-ATGCCAGTGAGCTTCCCGTTCAG-3); *C3* (forward: 5-CGCAACGAACAGGTGGAGATCA-3); reverse: 5-CTGGAAGTAGCGATTCTTGGCG-3); *C3AR1* (forward: 5-CTGGCGTAAAGATGAAGACGACC-3; reverse: 5-CCAGTGTCCTTGGAGAATCAGG-3); *GFAP* (forward: 5-CACCTACAGGAAATTGCTGGAGG-3; reverse: 5-CCACGATGTTCCTCTTGAGGTG-3) *IL-6* (forward: 5-CTCTGCAAGAGACTTCCATCCAGT-3; reverse: 5-GAAGTAGGGAAGGCCGTGG-3); *TNF-α* (forward: 5-AGGGTCTGGGCCATAGAACT-3; reverse: 5-CCACCACGCTCTTCTGTCTAC-3); and *TNFRSF1A* (forward: 5-GTGTGGCTGTAAGGAGAACCAG-3; reverse: 5-CACACGGTGTTCTGAGTCTCCT-3).

### Patient data analysis

Relevant patient data were acquired via the R2 database (https://hgserver1.amc.nl/cgi-bin/r2/main.cgi).

### Statistics

Experimental data were analyzed using GraphPad Prism software. Student’s *t* test was used to calculate the difference in experiments containing two groups. For experiments containing three or more groups, one-way ANOVA was used to determine the levels of difference, and the Bonferroni test was used to perform the posttests of all pairs of data. Differences were considered to be significant when their value was less than 0.05 (*P* < 0.05), while *P* < 0.01 was considered to be a very significant difference. Data are expressed as the mean ± SEM.

## Results

### Complement C3 is activated and releases C3a in tumor tissue during MB development

**Fig. 1:**
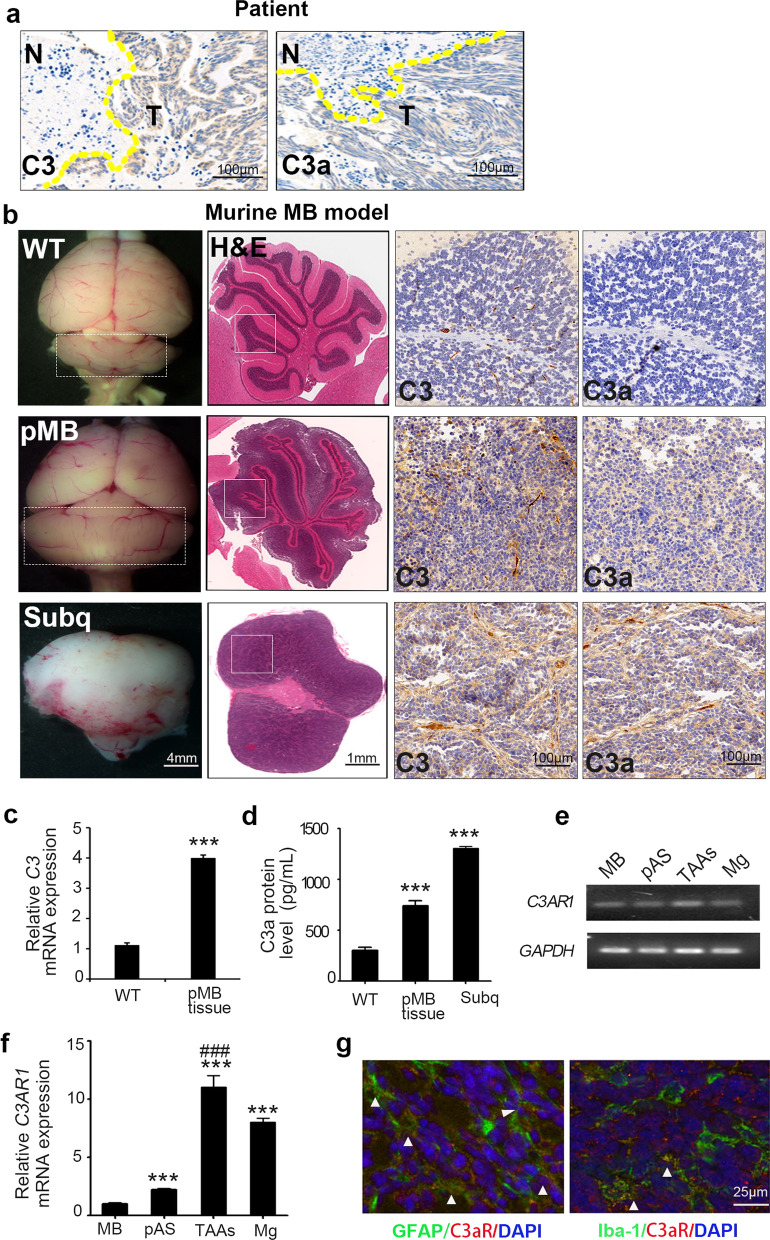
Complement C3a is present in MB tissue. Human and mouse MB sections underwent IHC staining for C3 and C3a. For detecting C3, anti-human/mouse C3 polyclonal antibody (GeneTex) was used, and for detecting C3a, the neoepitope recognizing antibodies were used as follows: anti-human C3a monoclonal antibody, clone 2991 (Bio-Rad) and anti-mouse C3a monoclonal antibody, clone I87-1162 (BD Biosciences). **a** Representative IHC staining of intact C3 (left) and C3a (right) in human Hh-type MB tissue; brown color displays the positive staining. *N* normal adjacent tissue; *T* tumor area. The yellow line defines the boundary between normal and tumor area. **b** Left upper and middle panels show the whole mount of WT normal brain and tumor-bearing brain of primary MB mouse (pMB), respectively, with normal cerebellum or MB tumor framed. The left lower panel shows the dissected tumor mass from the subcutaneously transplanted MB model (Subq). The 2nd panels show H&E staining of paraffin-embedded sections of the above murine MB tissue. Frames line out the area for subsequent IHC staining images. The 3rd and 4th panels show representative IHC staining of intact C3 and C3a in murine MB tumor tissue. **c** Quantitative PCR was performed to determine C3 mRNA levels in the normal cerebellum of WT mice and in primary MB tissue. **d** C3a protein levels in the WT normal cerebellum, primary MB tissue and subcutaneously transplanted MB tissue were assessed by ELISA. ***p < 0.001 vs. WT group in c and d. Conventional RT–PCR (**e**) and quantitative RT–PCR (**f**) were performed to detect C3a receptor (C3aR, gene: *C3AR1*) expression in primary astrocytes (pAS), tumor-associated astrocytes (TAAs), purified MB cells (MB) and mouse primary microglia (Mg); GAPDH served as an internal control. ***p < 0.001 vs. MB cells group and ### *p* < 0.001 vs. pAS group in **f**. **g** Frozen sections were prepared with primary murine MB tumor tissues, and immunostaining of C3aR (in red) and GFAP (in green) or Iba-1(in green) were performed to determine the C3aR distribution. Values are expressed as the mean ± SEM from at least three independent experiments in **c**, **d** and **f**. *(IHC*: immunohistochemistry)

To explore whether C3a, the complement system activation product, is involved in MB tumorigenesis, we first examined the presence of both intact C3 and its bioactive fragment C3a in MB tissue by IHC staining. As shown in Fig. 1a, tumor sections were obtained from Hh-type MB patients and stained for C3 and C3a proteins, and it was found that both C3 and C3a were abundantly present in the tumor area, whereas they were not detectable in the tumor-adjacent normal tissue area. C3a positive staining of more MB patient samples was shown in Additional file 1: Fig. S1a. Here, we used distinct antibodies for intact C3 or C3a detection: anti-C3 antibody recognizes the C-terminus of intact C3 (polyclonal antibody, GeneTex), while anti-C3a antibody (monoclonal antibody, clone 2991, Bio-Rad) can only react with the neoepitope of C3a after C3 cleavage but not intact C3. To observe this phenomenon in mice, a primary MB murine model based on transgenic mice (*Math1-Cre/Ptch1*^*loxp/loxp*^ mice) was utilized for investigation. In these transgenic mice, the Hh ligand antagonistic receptor *Patched 1* (*Ptch1*) is conditionally knocked out in Math1-positive cells. Math1 is the cell marker of granule neuron precursors (GNPs), which have been approved to be the Hh-type MB origin [41]. The absence of functional Ptch1 abolishes the inhibitory effect of Smoothened (Smo), which will in turn activate downstream transcription factors, such as glioma-associated oncogenes 1 and 2 (Gli1 and Gli2), followed by the aberrant activation of the Hh signaling pathway, resulting in MB formation. *Math1-Cre/Ptch1*^*loxp/loxp*^ mice develop MB spontaneously starting from 6 to 8 weeks with a 100% incidence rate. MB tumor tissues derived from *Math1-Cre/Ptch1*^*loxp/loxp*^ mice and normal cerebella of WT littermate controls were collected and sectioned for C3 and C3a protein detection by IHC. Similar to human patient samples, both intact C3 and C3a were distributed intensively in tumor tissue, whereas they were barely detected in the normal cerebella control (Fig. 1b, upper and middle panel). Since the subcutaneous transplantation MB model is widely used in basic and preclinical research, we also tested the presence of C3a in subcutaneous MB tissue, and C3/C3a showed a distribution pattern similar to that in primary intracranial MB tissue (Fig. 1b, lower panel). To further confirm local C3 expression in MB tissue, quantitative RT–PCR (qPCR) was performed to evaluate C3 mRNA levels. The results showed that compared with the WT normal cerebellum, primary MB tissue expressed much higher levels of C3 (Fig. 1c). In addition, by ELISA, C3a protein levels were found to be higher in both primary and transplanted MB tissues (Fig. 1d). The data above indicate that complement C3 is highly expressed and activated to release C3a in MB tissue, which prompted us to speculate that C3a might be involved in MB tumorigenesis. Moreover, a number of published studies have reported that C3a modulates astrocyte function in certain neuroinflammatory circumstances. Our previous work elucidated that TAAs are a crucial component of the MB tumor microenvironment favoring tumor development. We, therefore, hypothesize that C3a might be involved in MB progression by regulating TAA activation. As it is known that C3a plays its biological role by engaging the C3a receptor (C3aR), we then examined C3aR expression on TAAs and MB tumor cells isolated from *Math1-Cre/Ptch1*^*loxp/loxp*^ MB, as well as primary astrocytes derived from the normal cerebellum of WT neonatal mice by conventional PCR and qPCR. The murine primary cultured microglia served as the positive control for C3aR expression. In MB tumors, both tumor cells and TAAs expressed C3aR. In addition, TAAs expressed more C3aR than normal primary astrocytes (Fig. 1e, f). Moreover, immunostaining of C3aR on MB tumor sections also showed that C3aR was mainly expressed on astrocytes (GFAP +) as well as microglia (Iba-1 +) in murine (Fig. 1g) and human tumor tissue (Additional file 1: Fig. S1b). Thus, C3a is present in MB tissue, and its receptor is highly expressed on TAAs.

### C3a activates astrocytes, which promotes MB cell proliferation in vitro

**Fig. 2:**
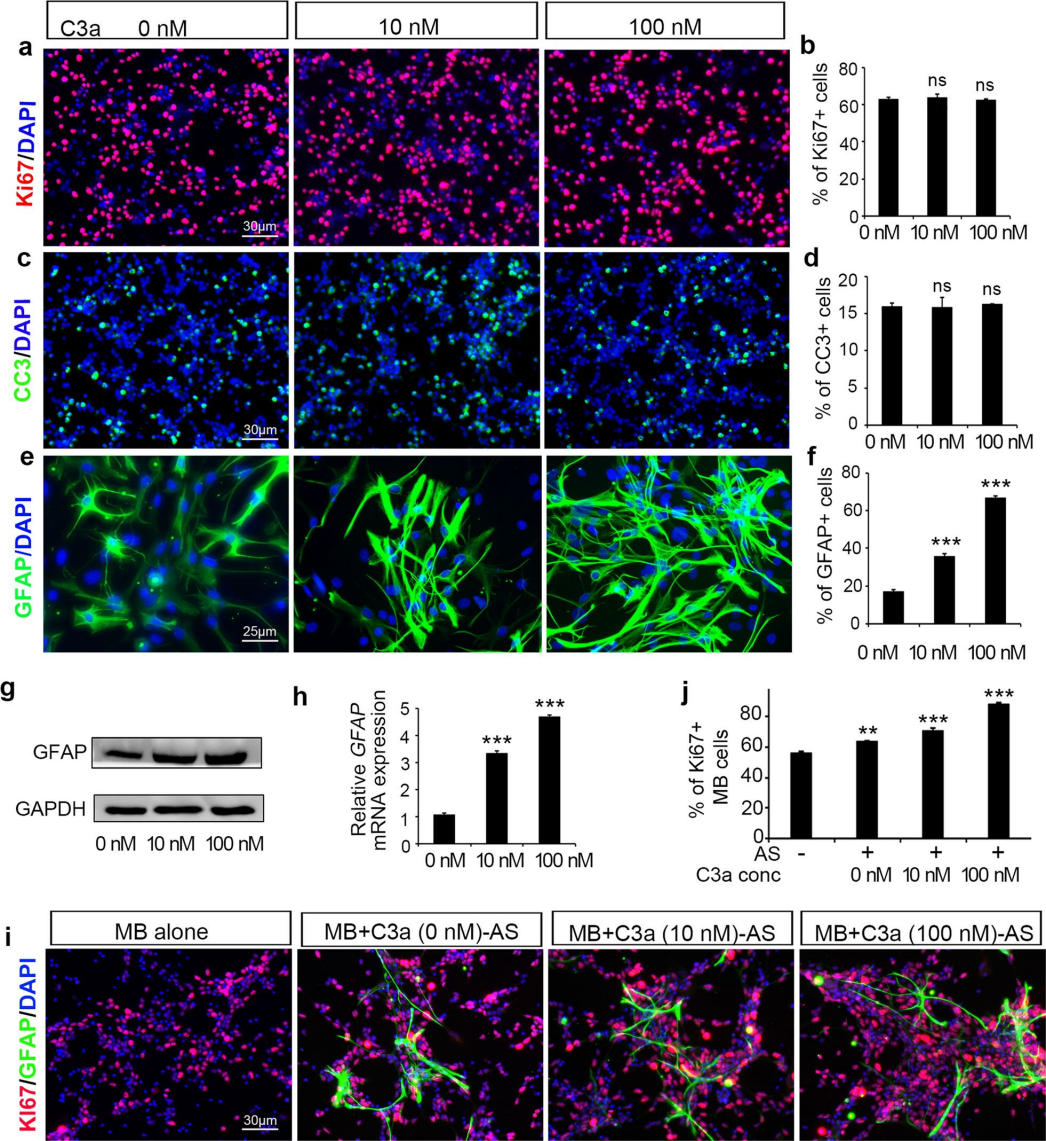
C3a activates astrocytes, promoting MB cell proliferation in vitro. **a**–**d** Purified primary MB cells were treated with C3a at the indicated concentration for 48 h in vitro. Immunostaining of Ki67 in red color (**a** and **b**) or cleaved caspase 3 (CC3, **c** and **d**) in green color was performed to determine the proliferation and apoptosis of MB cells. DAPI-counterstained cell nuclei are shown in blue color. Quantifications of the percentages of Ki67 + cells (**b**) and CC3 + cells (**d**) were plotted according to a and c, respectively. **e**–**f** Primary astrocytes were stimulated with C3a at the indicated concentration for 48 h in vitro. Immunostaining of GFAP (green) was performed to examine astrocyte activation (**e**), and the percentage of GFAP + cells was quantified (**f**). Astrocytes were also harvested after C3a administration for GFAP expression assessment by western blotting at the protein level (**g**) and qPCR at the mRNA level (**h**). GAPDH served as a protein sample loading control. **p < 0.01, ***p < 0.001 vs. (0 nM C3a) group in **b**, **d**, **f** and **h**. ns: not significant. **i**, **j** Astrocytes were pretreated with C3a at the indicated concentration for 24 h and washed thoroughly before being cocultured with MB cells at a 1:10 ratio for another 48 h. Then, the cells were immunostained with Ki67 and GFAP (**i**), and the percentage of Ki67 + /GFAP− cells was quantified (**j**). **p < 0.01, ***p < 0.001 vs. (MB alone) group in **j**. Images are representative, and values are expressed as the mean ± SEM from at least three independent experiments

Considering that both MB cells and astrocytes express C3aR, we wondered whether C3a affects tumor cell proliferation or astrocyte performance. To address this question, purified MB cells and normal primary astrocytes were cultured and treated with recombinant mouse C3a at the indicated concentrations in vitro (Fig. 2a–f). As shown in Fig. 2a, b, MB cell proliferation detected by immunostaining for the cell proliferation marker Ki67 was not altered by 48 h of treatment with C3a. In addition, the cleaved caspase 3 (CC3) level was not impacted by C3a treatment (Fig. 2c, d), indicating that C3a does not affect the proliferation or apoptosis of MB cells directly. Next, we investigated the effect of C3a on astrocytes. After treatment with C3a for 48 h in vitro, the astrocyte activation marker GFAP was assessed by immunofluorescent staining (Fig. 2e, f) and western blotting (Fig. 2g) at the protein level, as well as by qPCR at the mRNA level (Fig. 2h). We found that C3a markedly increased GFAP expression levels in astrocytes in a dose-dependent manner. Then, to verify our hypothesis that C3a may regulate MB cell proliferation indirectly by modulating astrocyte activation, we performed a coculture experiment of MB cells and astrocytes. Before coculture with MB cells, astrocytes were pretreated with incremental doses of C3a for 24 h and then harvested and washed thoroughly with PBS. After 48 h of coculture, the cells were fixed and immunostained for Ki67 to assess MB cell proliferation. As shown in Fig. 2i, j, MB cell proliferation was increased by coculture with astrocytes compared with MB cells alone; furthermore, pretreatment with C3a enhanced the ability of astrocytes to promote MB cell proliferation. Taken together, these results suggested that C3a could activate astrocytes, which increased the proliferation of MB tumor cells in vitro.

### C3a enhances TAA activation and MB tumor progression in vivo

**Fig. 3:**
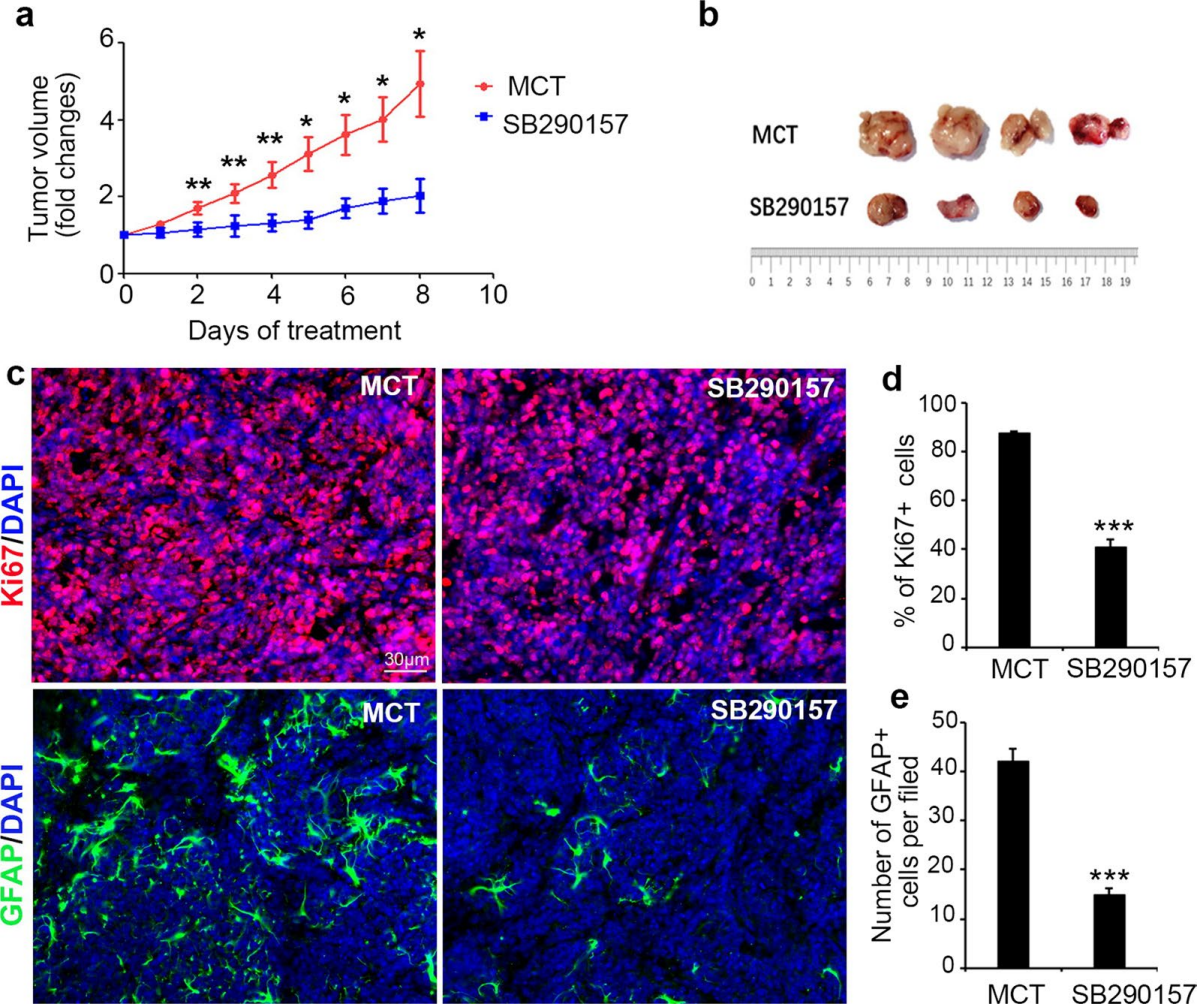
C3a enhances astrocyte activation and MB progression in vivo. A subcutaneous transplantation MB model was established to investigate the function of C3a in MB tumor growth in vivo. Whenever the subcutaneous tumor volume reached 200 mm^3^, tumor-bearing mice were treated with the C3aR antagonist SB290157 or the vehicle control (MCT) daily by intraperitoneal injection (30 mg/kg per mouse per day). Subcutaneous tumor volume was monitored every day, and the volume change was curved based on the fold changes (**a**), *p < 0.05, **p < 0.01. At the termination of the treatment experiment, tumors were dissociated and photographed to show tumor size (**b**). **c**–**e** Frozen sections were prepared with tumor tissues after treatment. Immunostaining of Ki67 (in red, upper panel in **c**) and GFAP (in green, lower panel in **c**) was performed to determine tumor cell proliferation and TAA activation, and DAPI was used to counterstain the cell nuclei. The percentage of Ki67 + cells (**d**) and the numbers of GFAP + cells per visual field (**e**) in tumor sections were quantified, ***p < 0.001 vs. the MCT treatment group. The results shown are representative of at least two independent experiments. Values are expressed as the mean ± SEM (*n* = 4 mice/group)

To further confirm the role of C3a in MB proliferation in vivo, a subcutaneous transplantation MB model was established. Briefly, primary MB tissue was dissociated from the cerebella of *Math1-Cre/Ptch1*^*loxp/loxp*^ mice and digested to obtain a single-cell suspension. These cells, including more than 90% MB cells, approximately 3–5% astrocytes and 5% microglia and oligodendrocytes [21, 22], were used to establish a subcutaneous transplantation model. Tumor-bearing mice were treated with a C3aR antagonist, SB290157, which is widely used in C3a studies to block the C3a–C3aR interaction. The activity of SB290157 was tested by inhibiting GFAP expression of C3a-treated astrocytes in vitro (Additional file 1: Fig. S2). Tumor volume was monitored every day starting from the first treatment, and the volume change was calculated and demonstrated a curved trend, as shown in Fig. 3a. By comparison with the vehicle (MCT) treatment group, 30 mg/kg C3aR antagonist daily treatment significantly attenuated tumor progression in vivo. We also tried lower doses of SB290157 (10 mg/kg) or a lower frequency of treatments, though there was no effect on tumor growth (data not shown). Therefore, we ceased performing these treatments in formal experiments. Then, MB xenografts were dissociated at the termination of treatment, and tumor size was compared. As shown in Fig. 3b, the tumor size was much smaller in the C3aR antagonist treatment group than in the control group. MB xenografts were also harvested for sectioning to detect MB cell proliferation and TAA activation status by immunostaining with Ki67 and GFAP, respectively. The results showed that the C3aR antagonist significantly reduced the percentage of proliferating tumor cells and the numbers of activating TAAs (Fig. 3c–e). These data demonstrated that C3a can enhance MB cell proliferation, TAA activation and tumor growth in vivo.

### C3a activates astrocytes via the p38 MAPK pathway

**Fig. 4:**
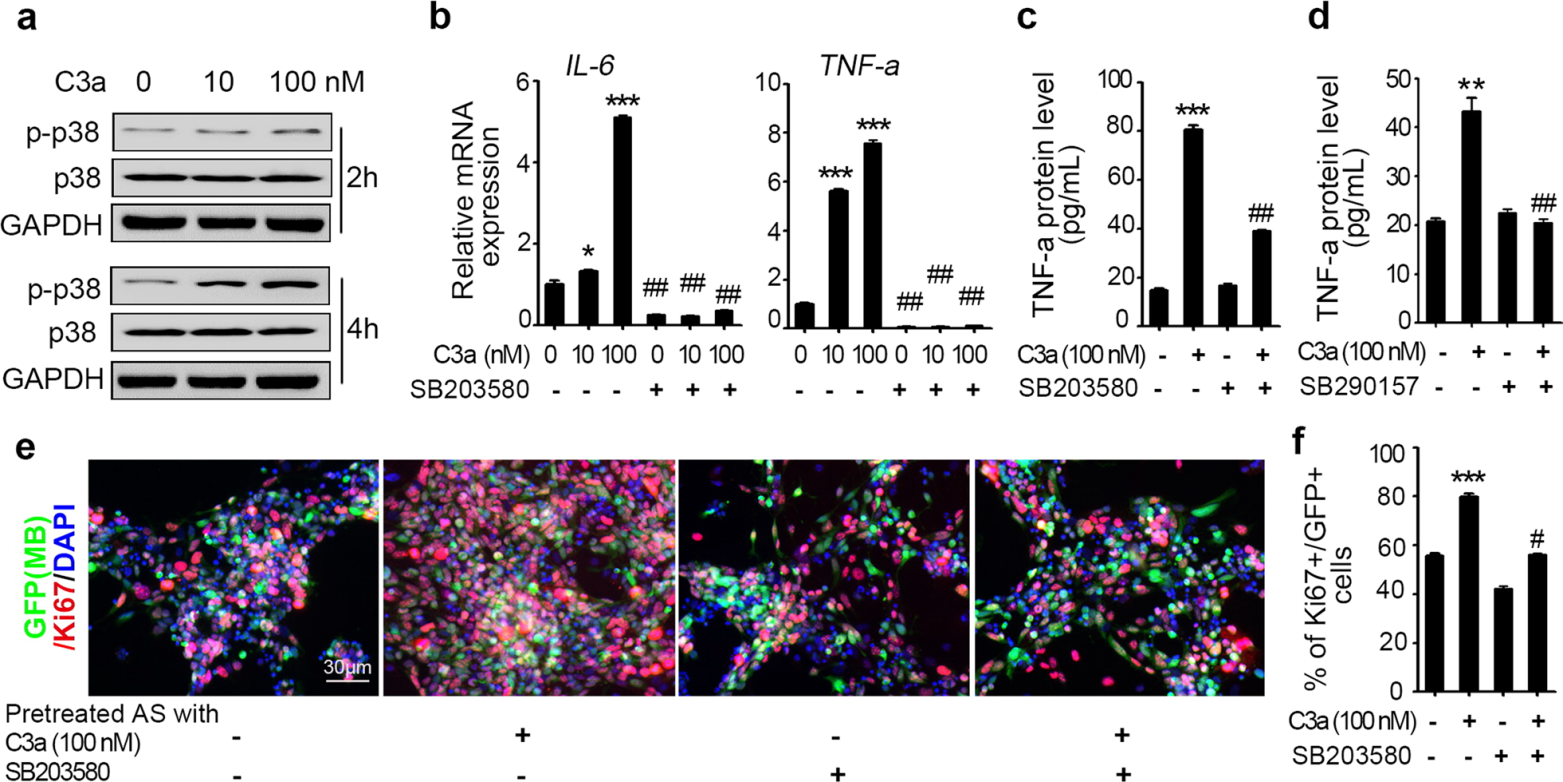
C3a activates astrocytes via the p38 MAPK pathway. **a** Primary astrocytes were cultured in vitro and stimulated with C3a at the indicated concentrations for 2 and 4 h. Then, the cells were harvested and lysed for total p38 and phosphorylated p38 (p-p38) detection by western blotting. GAPDH served as a protein sample loading control. **b**, **c** Astrocytes were administered with C3a at the indicated concentrations in the presence or absence of a p38 inhibitor SB203580 (2 μM) as indicated for 12 h (**b**) or 24 h (**c**). Then, the cells were harvested to perform qPCR for evaluations of IL-6 and TNF-α expression (**b**), or the conditioned medium was collected to assess the secretion of TNF-α protein by ELISA (**c**). **d** Astrocytes were administered with C3a (100 nM) in the presence or absence of a C3aR antagonist SB290157 (2 μM) for 24 h, then the conditioned medium was collected to assess the TNF-α production by ELISA, **p < 0.01 vs. C3a (−) & SB290157 (−) group, ##*p* < 0.01 vs. C3a ( +) & SB290157 (−) group. **e**–**f** Astrocytes were pretreated as above and washed thoroughly before coculture with MB cells derived from *Math1-Cre/Ptch1*^*loxp/loxp*^*/eGFP* mice at a 1:10 ratio. Forty-eight hours later, the cells were fixed to perform immunostaining for Ki67 (red). DAPI was used to counterstain the cell nuclei (**e**). The percentage of Ki67 + in GFP + cells was quantified (**f**). In b, c and f, *p < 0.05, ***p < 0.001 vs. C3a (−) & SB203580 (−) group; # *p* < 0.05, ##*p* < 0.01 vs. C3a ( +) & SB203580 (−) counterparts; ns: not significant. Images are presented, and values are expressed as the mean ± SEM from three independent experiments

C3a–C3aR is universally accepted to fulfill bioactivities mediated by the MAPK pathway [42, 43]. To determine whether C3a regulates astrocyte features through the MAPK pathway, we examined MAPK pathway activation in the astrocytes administrated with C3a. Using western blotting, we found that after 2 h of C3a administration, p38 phosphorylation was slightly upregulated in astrocytes. Until 4 h after administration, phosphorylated p38 was markedly increased in astrocytes even with a low concentration of C3a (10 nM), whereas the total amount of p38 was not changed by C3a administration (Fig. 4a). We also detected activation of the Erk pathway, while no activation was found based on C3a administration (Additional file 1: Fig. S3). This result suggested that C3a-triggered astrocyte activation might be mediated by the p38 MAPK pathway. Moreover, as a result of C3a–C3aR–MAPK pathway activation in astrocytes, some proinflammatory cytokines are produced, such as IL-6 and TNF-α [29]. We, therefore, examined the expression of these cytokines in astrocytes to further confirm whether the regulation of astrocytes by C3a was mediated by the p38 pathway. Primary astrocytes were cultured with C3a in the presence or absence of the p38 inhibitor SB203580 (2 µM). Twelve and 24 h after incubation, the cells were harvested for qPCR analysis, and conditioned medium (CM) was collected for ELISA analysis, respectively. As shown in Fig. 4b, C3a augmented *IL-6* and *TNF-α* expression in astrocytes in a dose-dependent manner, whereas the p38 inhibitor entirely counteracted the cytokine augmentation by C3a to an extent even lower than the basal level. We also evaluated TNF-α protein levels in C3a/p38 inhibitor-treated astrocyte CM. Likewise, C3a induced TNF-α secretion by astrocytes, and the induction was significantly impaired by p38 inhibition (Fig. 4c). Here, the TNF-α protein level in the C3a/SB203580 (2 μM)-treated group was not reduced to the basal level of the mRNA counterpart, possibly because the consumption of TNF-α protein was not completed at 24 h after incubation. Considering that some commercial recombinant C3a might have some nonspecific effect on the p38 pathway activation and cytokine production, to exclude the possibility that the astrocyte activation triggered by C3a might be due to the nonspecific effect of the recombinant C3a used in our experiments, we tested whether the TNF-α production in C3a-administrated astrocytes would be blocked by targeting C3a–C3aR signaling pathway. As shown in Fig. 4d, addition of C3aR antagonist SB290157 (2 μM) inhibited the TNF-α production by C3a-administrated astrocytes. In addition, the phosphorylation of p38 in C3a-admininstrated astrocytes was significantly reduced by SB290157 (Additional file 1: Fig. S4). Moreover, these experiments were repeated with recombinant C3a obtained from second company (R&D Systems) in the presence or absence of SB290157 (2 μM). The results showed that C3a from R&D Systems induced GFAP and TNF-α expression and the phosphorylation of p38, which could be inhibited by addition of SB290157 (Additional file 1: Fig. S5). The results above confirmed that C3a activated the p38 MAPK pathway and cytokine production in astrocytes through engaging C3aR in vitro. Furthermore, to confirm whether the tumor-promoting ability of C3a-activated astrocytes is mediated by the p38 pathway, a coculture experiment was performed. As described earlier, astrocytes were pretreated with C3a in the presence or absence of a p38 inhibitor for 24 h and then harvested and washed before being cocultured with MB cells derived from *Math1-Cre/Ptch1*^*loxp/loxp*^*/eGFP* mice for another 48 h. Immunostaining of Ki67 was performed, and Ki67 + MB cells (GFP +) were counted. As shown in Fig. 4e, f, C3a-pretreated astrocytes induced more MB cell proliferation, while the p38 inhibitor inhibited the effect of C3a. Furthermore, we tested p38 pathway activation in the tumor tissue of C3aR antagonist-treated subcutaneous MB mice and found that compared with vehicle treatment, C3aR antagonist treatment inhibited p38 pathway activation in vivo (Additional file 1: Fig. S6). These data indicate that C3a-triggered astrocyte activation is mediated by the p38 MAPK pathway.

### C3a activated astrocytes promote MB cell proliferation through TNF-α secretion

**Fig. 5:**
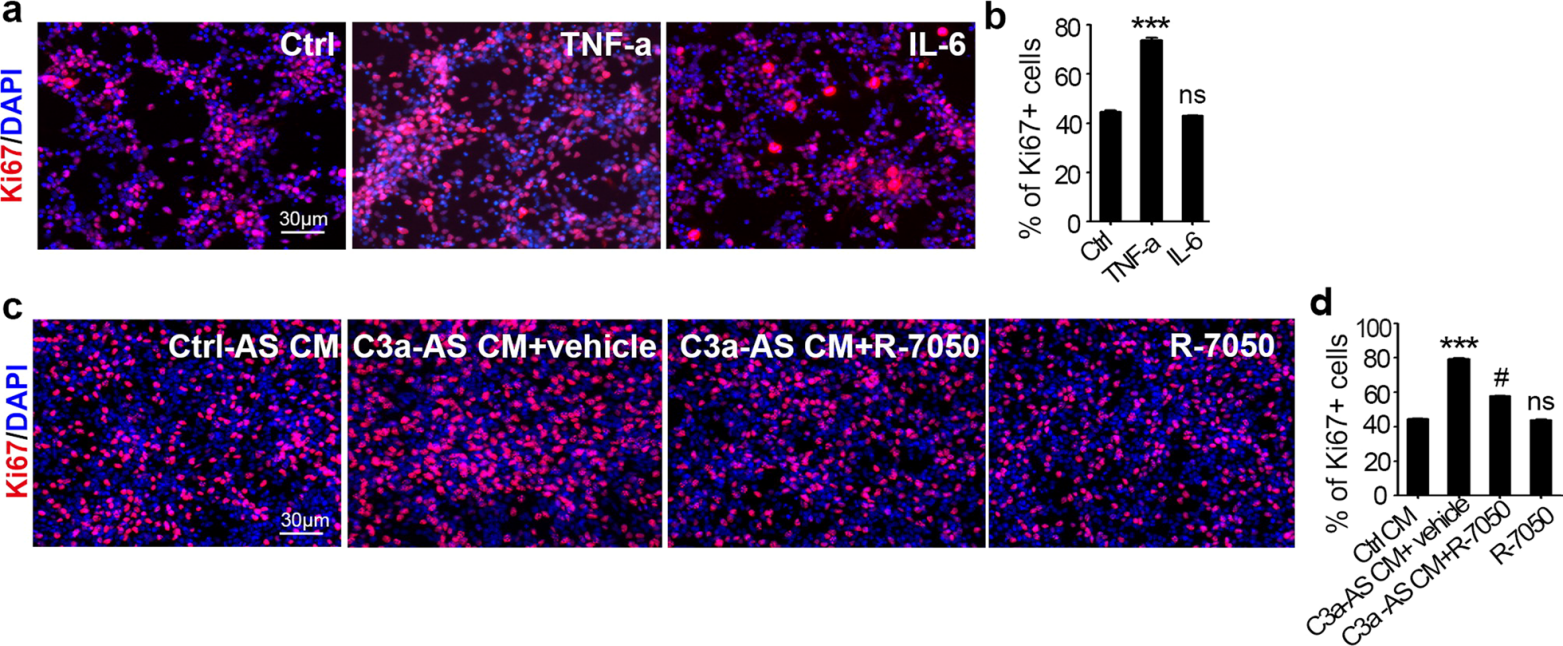
C3a-activated astrocytes promote MB cell proliferation through TNF-α secretion.** a**, **b** Purified MB cells were cultured in vitro in the presence of recombinant TNF-α, IL-6, or vehicle control (Ctrl) for 48 h and then immunostained for Ki67 (red). DAPI was used to counterstain the cell nuclei (**a**). The percentage of Ki67 + cells was quantified (**b**). ***p < 0.001, ns: not significant vs. Ctrl group. **c**, **d** Astrocytes were pretreated with 100 nM C3a or vehicle control for 24 h, washed thoroughly, and cultured for another 24 h for CM collection. Then, purified MB cells were cultured with the CM above together with the TNF-α receptor antagonist R-7050 (500 nM) or not for 48 h. Ki67 was immunostained (**c**), and the percentage of Ki67 + cells was quantified (**d**). ***p < 0.001, ns: not significant vs. Ctrl CM group, # *p* < 0.05 vs. (C3a-AS CM + vehicle) group. Images are presented, and values are expressed as the mean ± SEM from three independent experiments

Next, we explored the mechanism by which C3a-activated astrocytes regulate MB tumor cell proliferation. Our previous work demonstrated that TAAs could produce Hh ligands to maintain Hh signaling in MB cells via the noncanonical Hh pathway [21]; therefore, we first tested whether C3a-activated astrocytes could express more Hh ligands; however, no significant increase in Hh mRNA in astrocytes was found after C3a treatment in vitro (data not shown). Then, we investigated the molecules that had been verified to be highly expressed in astrocytes attributed to C3a stimulation in our study: GFAP, TNF-α and IL-6. Among them, TNF-α was proven by some studies to accelerate tumor growth [44, 45], and we then examined whether it could also enhance MB cell proliferation. First, we confirmed TNF-α receptor (TNF-αR) expression on MB cells (Additional file 1: Fig. S7). Then we stimulated purified MB cells with recombinant TNF-α (500 nM) for 48 h. IL-6 (500 nM) was also included for parallel experiments. Ki67 staining was used to evaluate the proliferation of MB cells. As shown in Fig. 5a, b, TNF-α but not IL-6 significantly enhanced MB cell proliferation compared with the vehicle control. Subsequently, to determine whether C3a-activated astrocytes promoted MB cell proliferation through TNF-α production, we collected astrocyte CM to perform the experiment. Primary astrocytes were pretreated with C3a or vehicle control for 24 h and washed thoroughly. Then, astrocytes were cultured for another 24 h without C3a to obtain CM. Next, MB cells were cultured with the above astrocyte CM in the presence or absence of the TNF-αR antagonist R-7050 (500 nM) for 48 h. Ki67 was detected by immunofluorescence, and Ki67 + cells were counted for MB cell proliferation evaluation. As shown in Fig. 5c, d, compared with control CM, CM of C3a-pretreated astrocytes markedly increased the proliferation of MB cells, whereas the TNF-αR antagonist R-7050 significantly abolished this effect. To exclude the possible influence of R-7050 on MB proliferation, MB cells were also cultured with a single administration of R-7050, and no difference in proliferation was detected compared with the control group. The above results elucidate that C3a-activated astrocytes promote MB cell proliferation through TNF-α secretion.

### TNF-α facilitates MB tumor progression in vivo

**Fig. 6:**
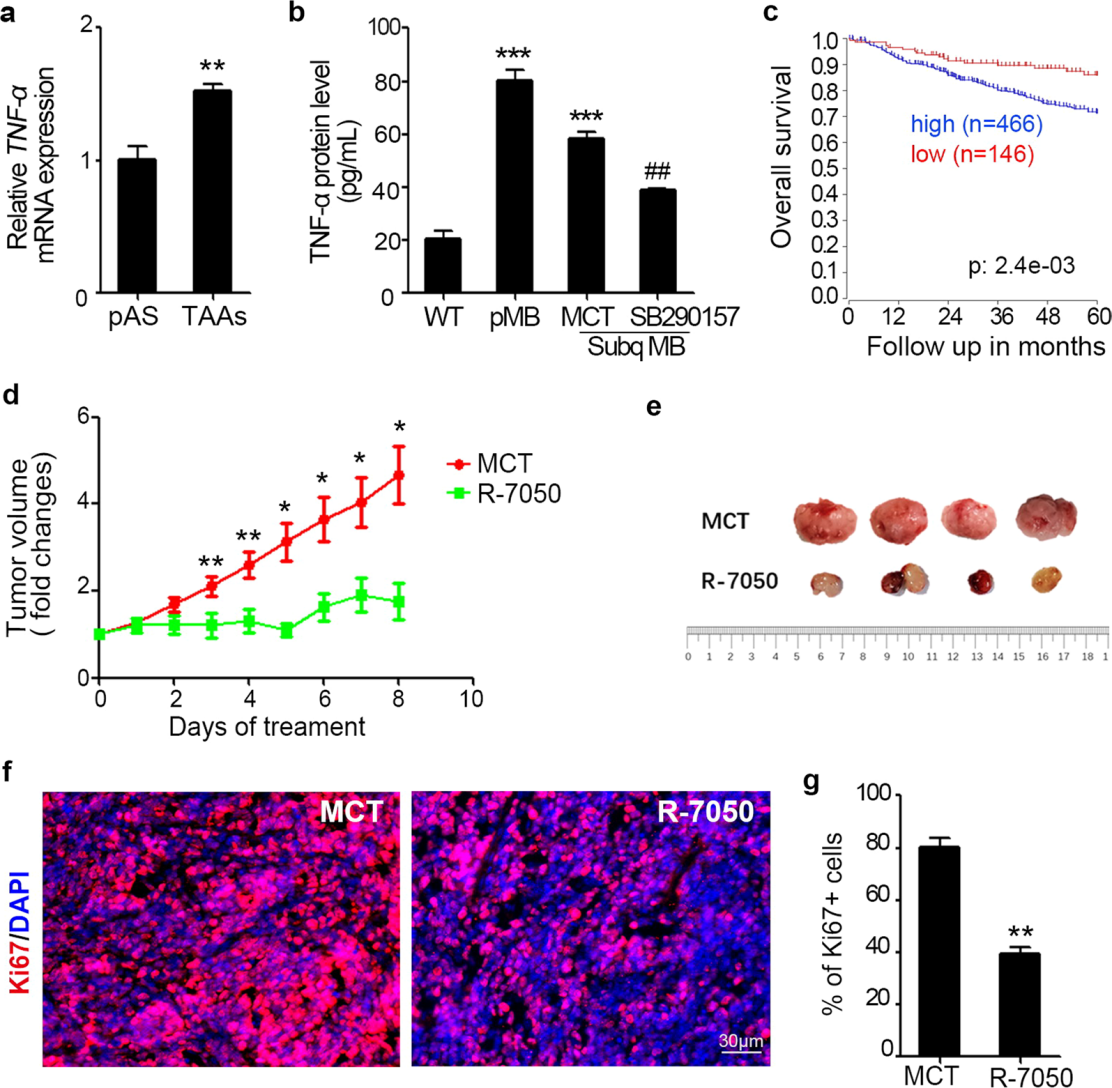
TNF-α facilitates MB tumor progression in vivo. **a** Primary astrocytes from the normal cerebella of WT mice (pAS) and TAAs were isolated and prepared for TNF-α mRNA evaluation by qPCR, **p < 0.01. **b** Normal cerebella of WT mice (WT), primary MB tissue and subcutaneous MB tissue treated with the C3aR antagonist SB290157 or MCT control were harvested and homogenized in lysis buffer, and the tissue homogenate samples were centrifuged to collect the supernatant. Then, TNF-α protein levels in these supernatants were assessed by ELISA. ***p < 0.001 vs. WT group; ##*p* < 0.01 vs. MCT group. **c** Overall survival rate of MB patients in TNF-αR high (blue line) and low (red line) expression groups was followed up to 60 months. Analysis was performed using the R2 Genomics Analysis and Visualization Platform. **d**–**g** Subcutaneous MB-bearing mice were treated with the TNF-α antagonist R-7050 (30 mg/kg per mouse per day) or vehicle control (MCT) daily by intraperitoneal injection. Subcutaneous tumor volume was monitored every day, and the volume was curved based on the fold changes (**d**). At the termination of treatment, tumors were dissociated and photographed to show tumor size (**e**). Frozen sections were prepared from tumor tissues after treatment. Ki67 was immunostained red, and DAPI was used to counterstain the cell nuclei (**f**). The percentage of Ki67 +  cells in tumor sections was quantified (**g**), **p < 0.01. The results shown are representative of two independent experiments. Values are expressed as the mean ± SEM (*n* = 4 mice/group)

Furthermore, we investigated whether TNF-α production induced by C3a could contribute to MB growth in vivo. We first compared TNF-α mRNA expression in normal primary astrocytes and TAAs, it was found that TAAs expressed more TNF-α (Fig. 6a). We then examined TNF-α protein levels in MB tissues, including primary intracranial MB, subcutaneously transplanted MB treated with C3aR antagonist or MCT control. The results showed that there was much more TNF-α protein accumulation in both primary and subcutaneous MB tissues compared with the WT normal cerebellum (Fig. 6b) and that when the subcutaneous MB-bearing mice were treated with the C3aR antagonist SB290157, the TNF-α protein level in tumor tissue was reduced significantly compared with the MCT-treated group (Fig. 6b), suggesting that TNF-α might be involved in C3a’s regulation of MB development in vivo. Since we determined the positive expression of TNF-αR on MB cells, we next consulted the “R2” Genomics Analysis and Visualization Platform to analyze the correlation between TNF-αR mRNA expression levels of tumors in MB patients and their prognosis. A total of 632 cases were analyzed, including 466 patients with high TNF-αR expression and 146 patients with low expression. As shown in Fig. 6c, up to 60 months, the overall survival rates were analyzed, and the prognosis in the TNF-αR low expression group was significantly better than that in the high expression group, which suggested that the TNF-αR signaling pathway is harmful to MB development. To verify the role of TNF-α in MB development in vivo, treatment with a TNF-αR antagonist was performed by utilizing a transplantation MB model. We tested a smaller dose of R-7050 for treatment in a preliminary experiment and chose the dose of 30 mg/kg body weight per mouse once a day. As shown in Fig. 6d, compared with the control MCT-treated group, TNF-αR antagonist treatment effectively impeded tumor progression. MB xenografts were dissected after 8 days of treatment to compare the tumor size, which was much smaller in the TNF-αR antagonist treatment group (Fig. 6e). Simultaneously, tumor tissue was sectioned to examine tumor cell proliferation by Ki67 immunostaining. As shown in Fig. 6f, g, TNF-αR antagonist treatment distinctly dampened MB cell proliferation in vivo. These data indicate that TNF-α promotes MB tumor growth in vivo.

## Discussion

Current therapies for MB are far from optimal. Patients always suffer from side effects caused by surgery combined with radio- and chemotherapy. Targeted drugs have been developed in the past decade; however, some drugs still show defects. For example, Hh signaling pathway inhibitors targeting SMO, vismodegib and sonidegib, have been reported to impact skeletal growth in pediatric patients, since Hh signaling is also required for bone development [9, 46, 47]. Moreover, treatment of MB or basal cell carcinoma (BCC), another Hh pathway-driven cancer, with these inhibitors causes gene mutations, including SMO and its downstream molecules, in tumor cells, which results in drug resistance [48–51]. However, unlike tumor cells, stromal cells in the TME show more genetic stability, so they present promising therapeutic targets for tumor treatment. Our previous publications demonstrated that astrocytes are an important TME component and are activated to play a critical role in MB. In the current study, we investigated the mechanisms of astrocyte activation and modification in MB development.

Here, we described that C3, the central factor of the complement system, was activated and released C3a in MB, and consistent with other publications, C3a activated astrocytes in vitro: C3a upregulated the expression of the astrocyte activation marker GFAP and some proinflammatory cytokines; C3a stimulated p38 MAPK pathway signal transduction in astrocytes; and C3a enhanced the tumor-promoting ability of astrocytes. Furthermore, C3aR antagonist treatment successfully suppressed astrocyte activation and MB progression in vivo, indicating that C3a contributes to MB development by activating astrocytes. We cannot exclude the involvement of other cells in the MB TME, such as microglia/macrophages, given that microglia/macrophages in the brain express C3aR [52, 53], and that C3a–C3aR signaling mediates microglial regulation of neuroinflammation in several neural system diseases. For example, C3a–C3aR signaling in microglia contributes to β-amyloid pathology and neuroinflammation in Alzheimer's disease [54], and this signaling in microglia was recently reported to drive the pathogenesis of neuromyelitis optica [55]. Meanwhile, microglia/macrophages were demonstrated to participate in the MB TME [56], although the conclusions are paradoxical: Maxmov et al. found that tumor-associated macrophages impair MB growth [57], whereas Yao et al. demonstrated that microglia promote MB progression through IGF1 production [58]. In our study, the ideal approach to exclude C3a’s effect on microglia/macrophages during MB development is to conditionally knock out C3aR in astrocytes of MB mice. That means to generate transgenic mice by crossmating *GFAP-cre* and *C3aR*^*loxp/loxp*^ mice, and then crossmate these mice with MB mice. Unfortunately, our MB model is based on *Math1-cre*/*Ptch1 *^*loxp/loxp*^, which makes it infeasible to further manipulate mouse breeding.

The function of C3a in medulloblastoma has seldom been reported. Maurer et al. found that knockdown of C3aR in Daoy cells, a human MB cell line, by siRNA reduced Daoy cell proliferation in vitro [59]. Inconsistently, in our study, although C3aR was expressed on MB cells, we found that C3a did not directly alter MB cell proliferation or apoptosis in vitro. In our coculture experiments, to minimize the effect of C3a on MB cells, C3a-pretreated astrocytes were washed thoroughly before coculture with MB cells, and astrocyte-conditioned medium was collected after removing C3a for another 24 h of culture. The reason why we obtained different results might lie in the difference between the immortalized human MB cell line and primary freshly harvested mouse MB cells. C3a is the smaller fragment released from intact C3 following complement activation, and C3 is widely expressed by somatic and myeloid cells. We observed that both intact C3 and C3a peptide levels were dramatically higher in MB tumor tissue than in adjacent normal tissue, indicating that C3 is activated during MB development. We did not identify the cell source of C3 in MB tumors considering that both C3 and C3a can be secreted into the fluid phase and play their roles in autocrine or paracrine manners. Astrocytes have been proven to express C3, and astrocyte-derived C3 regulates neuroinflammation in both Alzheimer's disease [54] and EAE [60] animal models. Thus, C3 in MB is probably provided by TAAs in our model. Regarding how C3 is activated in MB tumorigenesis, the question remains elusive. A recently published study demonstrated that C3 is activated through the lectin pathway during sarcoma progression [61]. An earlier study showed that the classical and lectin pathways but not alternative pathway are involved in TC-1 tumor growth in a subcutaneous cervical cancer model [62]. Further investigation is needed to explore the mechanism of C3 activation in MB.

Then, in the current study, we further investigated the mechanisms of astrocyte activation by C3a and their promotion of MB cell proliferation. We found that C3a triggered p38 MAPK pathway activation and TNF-α production in astrocytes; blockage of the p38 pathway in astrocytes or the TNF-αR pathway in MB cells significantly decreased MB cell proliferation in the coculture system, indicating that astrocytes activated by C3a promote MB cell proliferation by secreting TNF-α. TNF-α and other proinflammatory cytokines have been proven to be expressed based on C3a–C3aR signal transduction, and TNF-α is reported to have controversial roles in cancers; several studies have shown that it has protumor functions [44, 45]. Here, we found that MB cells expressed TNF-αR, and TNF-α directly enhanced MB cell proliferation; TNF-αR antagonist administration deprived MB cells of responding to conditioned medium from C3a pretreated astrocytes. Finally, TNF-αR antagonist treatment significantly retarded MB progression in vivo. Our data add new evidence for the tumor-promoting role of TNF-α. Given that many activation products might be induced in astrocytes by C3a–C3aR signaling, we attempted to knock down TNF-α by lentivirus carrying TNF-α shRNA in astrocytes and then performed the coculture experiments. However, even after infection with the control scrambled lentivirus, primary astrocytes almost lost their ability to increase MB cell proliferation in the coculture system. Therefore, we cannot absolutely exclude the effect of other molecules on MB promotion in the current system.

Hh-type MBs are driven by aberrant activation of Hh signaling, and our previous work demonstrated that astrocytes provide Hh ligands to maintain Hh signaling in MB cells. However, in the current study, we did not find that Hh ligand mRNA was remarkably upregulated in astrocytes after C3a stimulation. Meanwhile, neither C3aR nor TNF-αR antagonist treatment in vivo impacted Hh signaling pathway target gene expression in MB tumors (data not shown). These data suggest that C3a-triggered astrocyte TNF-α secretion dose not promote MB tumor growth by targetting Hh signaling. It seems that there is no crosstalk between the C3aR or TNF-αR and Hh signaling pathways in our model. Further efforts are needed to address these questions.

## Conclusions

**Fig. 7:**
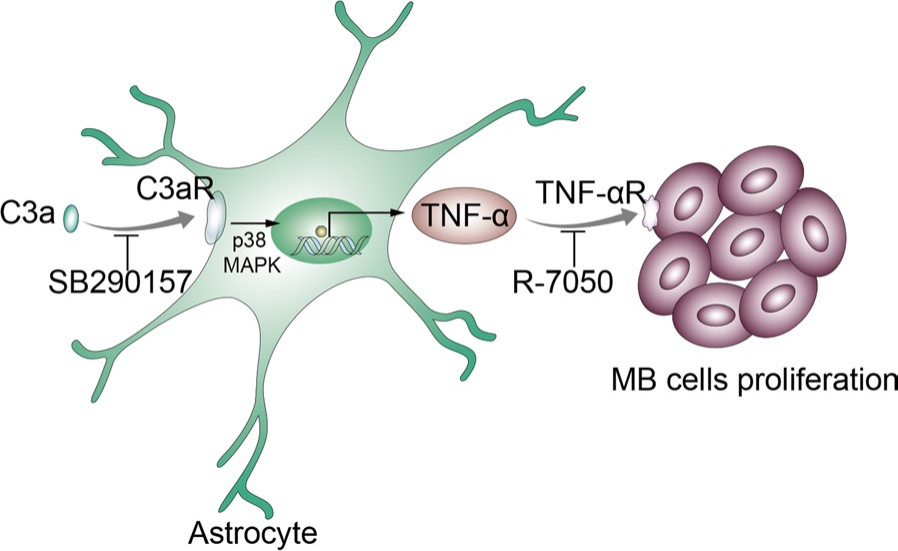
Graphic illustration of the conclusions

Collectively, our studies demonstrate that complement is activated in MB development, releasing C3a, which then induces TNF-α production in astrocytes via the p38 pathway, and TNF-α in turn promotes MB progression (Fig. 7). For the first time, we reveal complement’s role in astrocyte modification in MB TME, which contributes to MB progression. Our investigation also tested the feasibility of C3aR and TNF-αR antagonists in MB treatment, helping to provide new therapeutic targets and develop new therapeutic strategies for MB.

## Supplementary Information

**Additional file 1: Fig. S1.** C3a is present in the tumor tissue of MB patients and C3aR is present on both astrocytes and microglia. a, IHC staining of C3a on human MB sections derived from 4 patients, respectively (P#2–P#5). b, Representative immunostaining of C3aR (in red) and GFAP (in green) or Iba-1(in green) on human MB sections. **Fig. S2.** C3aR antagonist SB290157 inhibits GFAP expression by C3a-administrated astrocytes. Primary astrocytes were stimulated with C3a (100 nM) with or without SB290157 (2 μΜ) for 48 h in vitro. Immunostaining of GFAP (green) was performed (a), and the percentage of GFAP + cells was quantified (b). **Fig. S3.** C3a does not activate the Erk pathway in astrocytes. Primary astrocytes were stimulated with 100 nM C3a for 2, 5, 10 and 20 min. Then, the cells were harvested and lysed for total Erk and phosphorylated Erk (p-Erk) detection by western blotting. β-Tubulin served as a protein sample loading control. **Fig. S4.** Phosphorylation of p38 in C3a-administrated astrocytes is inhibited by C3aR antagonist SB290157. a, Primary astrocytes were stimulated with C3a (100 nM) with or without addition of SB290157 (2 μΜ) for 4 h in vitro. Then, the cells were harvested and lysed for total p38 and phosphorylated p38 (p–p38) detection by western blotting. **Fig. S5.** GFAP and TNF-α expression and the phosphorylation of p38 in C3a-administrated astrocytes is inhibited by C3aR antagonist SB290157. Primary astrocytes were stimulated with C3a (100 nM, from R&D) in the presence or absence of SB290157 (2 μΜ) for 48 h (a and b), 4 h (c) and 12 h (d), respectively, in vitro. a, Immunostaining of GFAP (in green) was performed, and b, the percentage of GFAP + cells was quantified. c, the cells were harvested and lysed for total p38 and phosphorylated p38 (p–p38) detection by western blotting. d, the cells were harvested to perform qPCR for evaluating TNF-α mRNA levels. ***p < 0.001 vs. C3a (−) & SB290157 (−) group, ##*p* < 0.01 and ###*p* < 0.001 vs. C3a ( +) & SB2901570 (−) group. **Fig. S6.** C3aR antagonist treatment inhibits the p38 MAPK pathway activation in tumor tissue of subcutaneous MB mice. Tumor tissue of C3aR antagonist (SB290157)- and vehicle (MCT)-treated subcutaneous MB mice was harvested at the termination of treatment, and tumor tissue was homogenized to evaluate phosphorylated p38 (p–p38) and total p38 levels by western blotting. β-Tubulin served as a protein sample loading control. **Fig. S7.** TNF-α receptor is expressed on MB cells. Primary MB cells (pMB), TAAs and BV2 cells were harvested and lysed for RT–PCR assays to detect TNF-α receptor (*TNFSF1A*) expression. GAPDH served as an internal control.

## Data Availability

The data that support the findings of this study are available from corresponding author on reasonable request.

## Abbreviations

APC: Allophycocyanin
IL: Interleukin
TNF: Tumor necrosis factor
PBS: Phosphate buffered saline
TBS: Tris-buffered saline
