# Supplementary material for: Complement C3a activates astrocytes to promote medulloblastoma progression through TNF-α
Source: J Neuroinflammation. 2022 Jun 20;19:159. doi: 10.1186/s12974-022-02516-9 (PMC9208237; doi:10.1186/s12974-022-02516-9)
Supplement: Supplementary file 1 — Additional file 1: Fig. S1. C3a is present in the tumor tissue of MB patients and C3aR is present on both astrocytes and microglia. a, IHC staining of C3a on human MB sections derived from 4 patients, respectively (P#2–P#5). b, Representative immunostaining of C3aR (in red) and GFAP (in green) or Iba-1(in green) on human MB sections. Fig. S2. C3aR antagonist SB290157 inhibits GFAP expression by C3a-administrated astrocytes. Primary astrocytes were stimulated with C3a (100 nM) with or without SB290157 (2 μΜ) for 48 h in vitro. Immunostaining of GFAP (green) was performed (a), and the percentage of GFAP + cells was quantified (b). Fig. S3. C3a does not activate the Erk pathway in astrocytes. Primary astrocytes were stimulated with 100 nM C3a for 2, 5, 10 and 20 min. Then, the cells were harvested and lysed for total Erk and phosphorylated Erk (p-Erk) detection by western blotting. β-Tubulin served as a protein sample loading control. Fig. S4. Phosphorylation of p38 in C3a-administrated astrocytes is inhibited by C3aR antagonist SB290157. a, Primary astrocytes were stimulated with C3a (100 nM) with or without addition of SB290157 (2 μΜ) for 4 h in vitro. Then, the cells were harvested and lysed for total p38 and phosphorylated p38 (p–p38) detection by western blotting. Fig. S5. GFAP and TNF-α expression and the phosphorylation of p38 in C3a-administrated astrocytes is inhibited by C3aR antagonist SB290157. Primary astrocytes were stimulated with C3a (100 nM, from R&D) in the presence or absence of SB290157 (2 μΜ) for 48 h (a and b), 4 h (c) and 12 h (d), respectively, in vitro. a, Immunostaining of GFAP (in green) was performed, and b, the percentage of GFAP + cells was quantified. c, the cells were harvested and lysed for total p38 and phosphorylated p38 (p–p38) detection by western blotting. d, the cells were harvested to perform qPCR for evaluating TNF-α mRNA levels. ***p < 0.001 vs. C3a (−) & SB290157 (−) group, ##p < 0.01 and ###p < 0.001 vs. C3a ( +) & SB2901570 [file 12974_2022_2516_MOESM1_ESM.pdf]

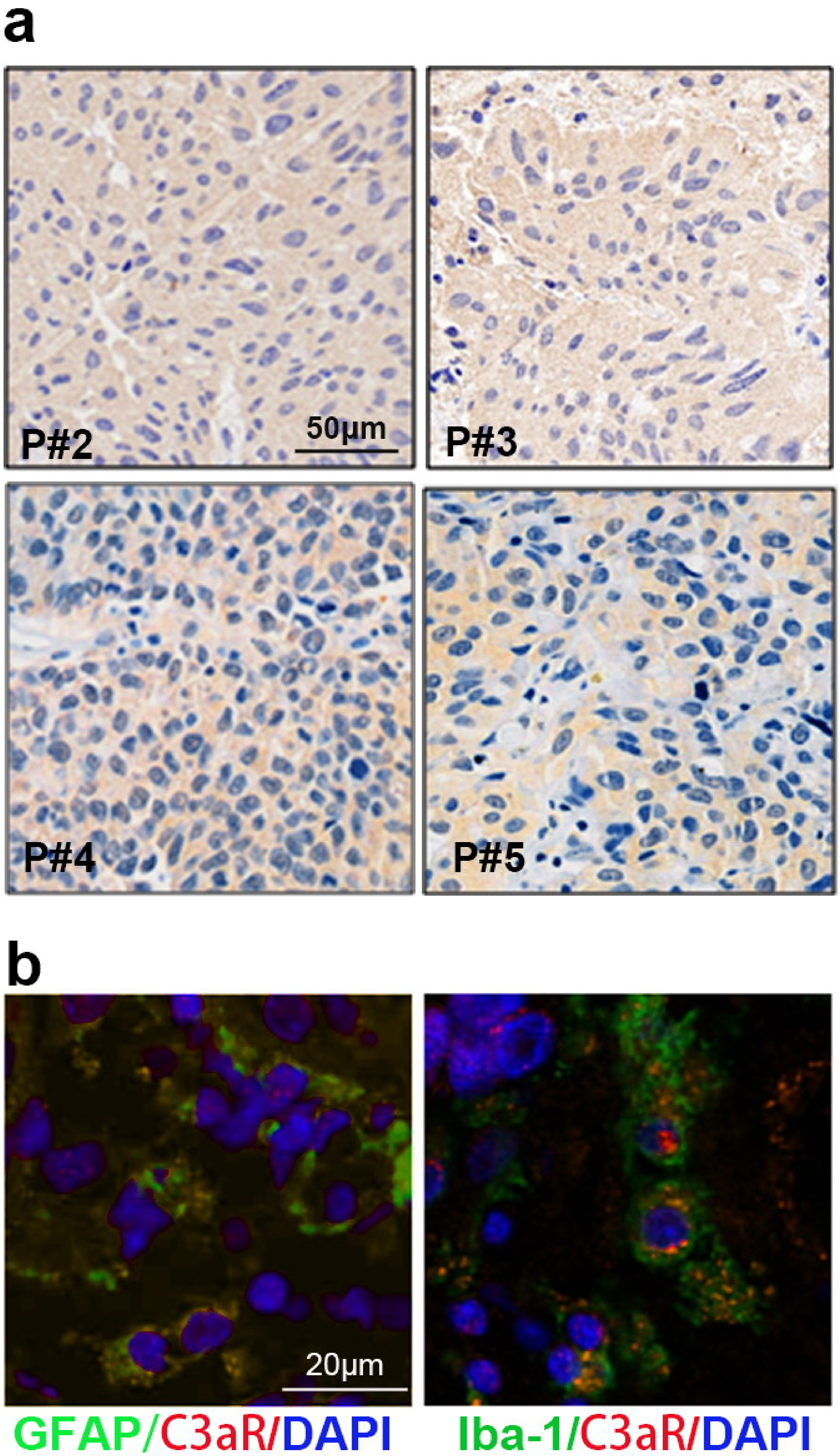

Supplementary Fig.1 C3a is present in the tumor tissue of MB patients and C3aR is expressed on TAAs and microglial

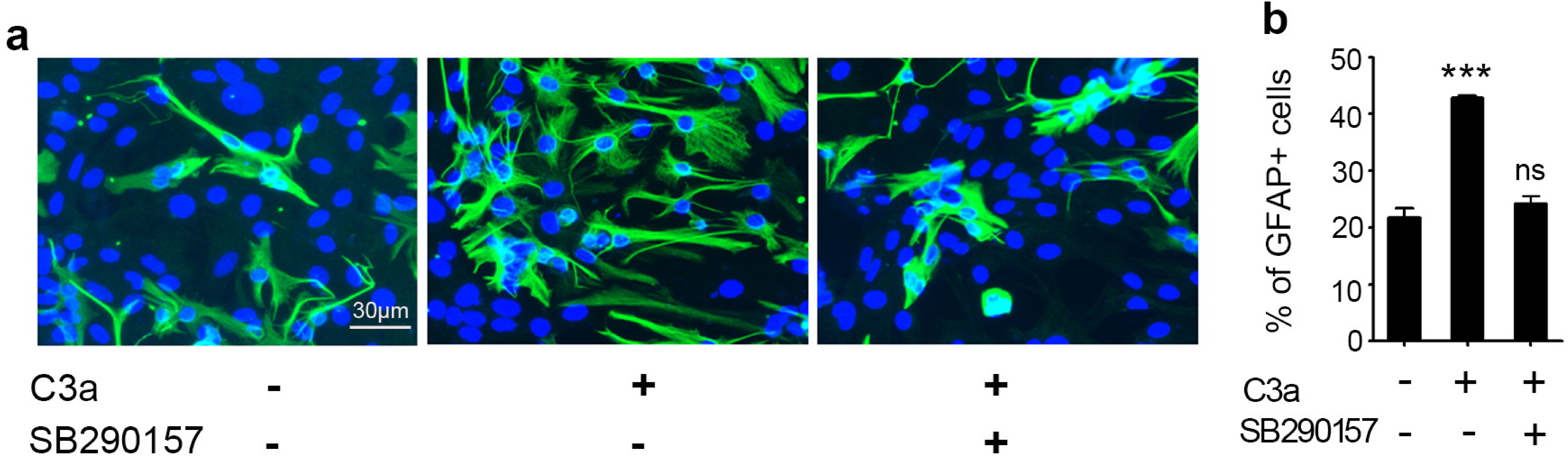

Supplementary Fig. 2 C3aR antagonist SB290157 inhibits GFAP expression by C3a-administrated astrocytes

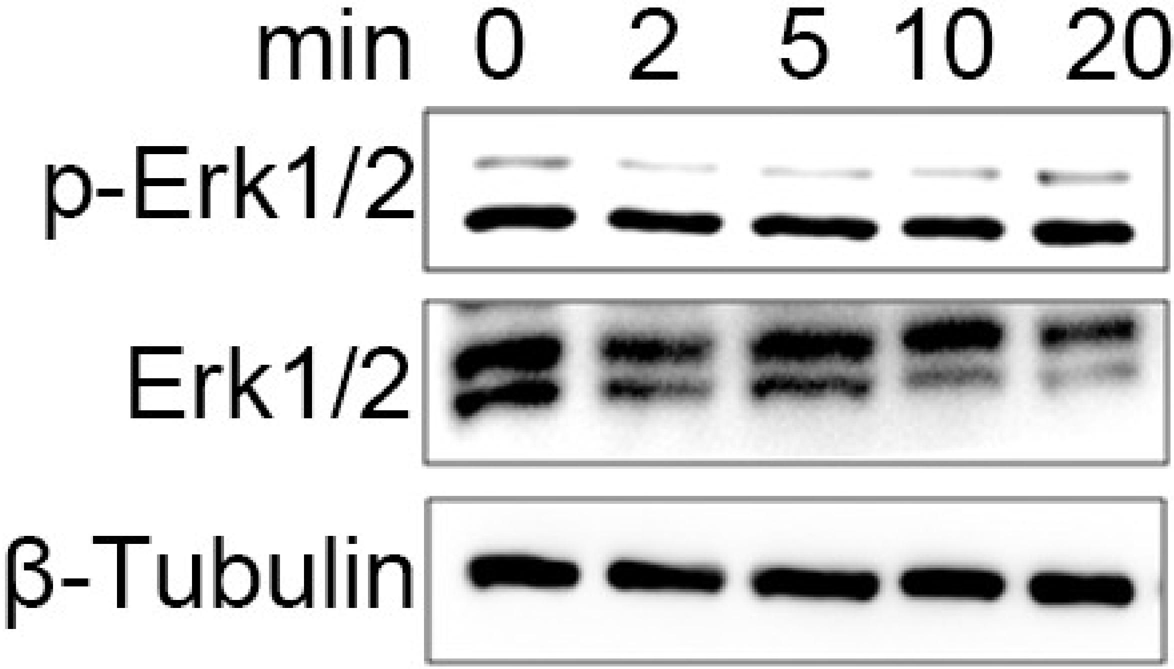

Supplementary Fig.3 C3a does not activate the Erk signal pathway in astrocytes

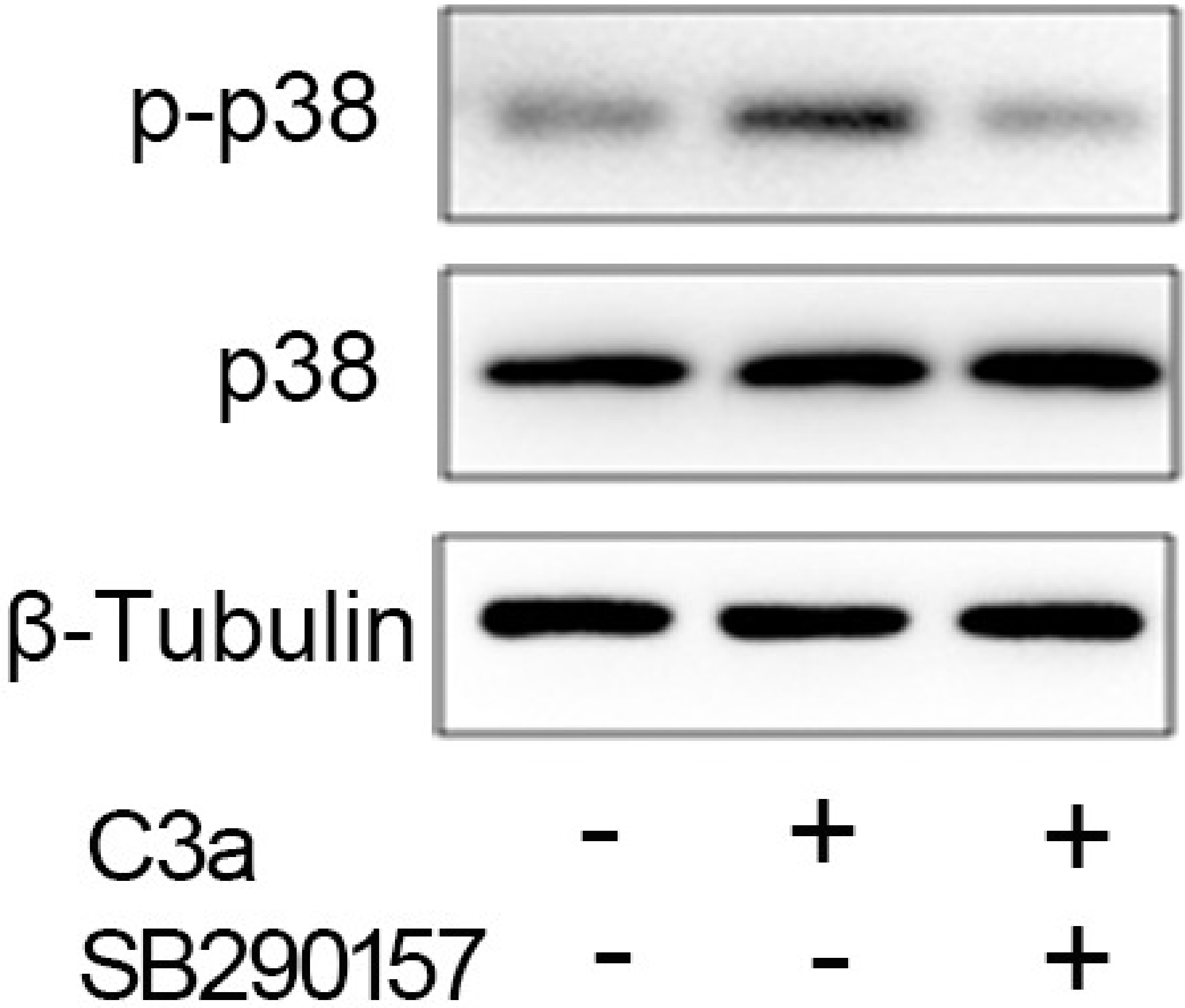

Supplementary Fig. 4 The phosphorylation of p38 in C3a-administrated astrocytes is inhibited by C3aR antagonist SB290157

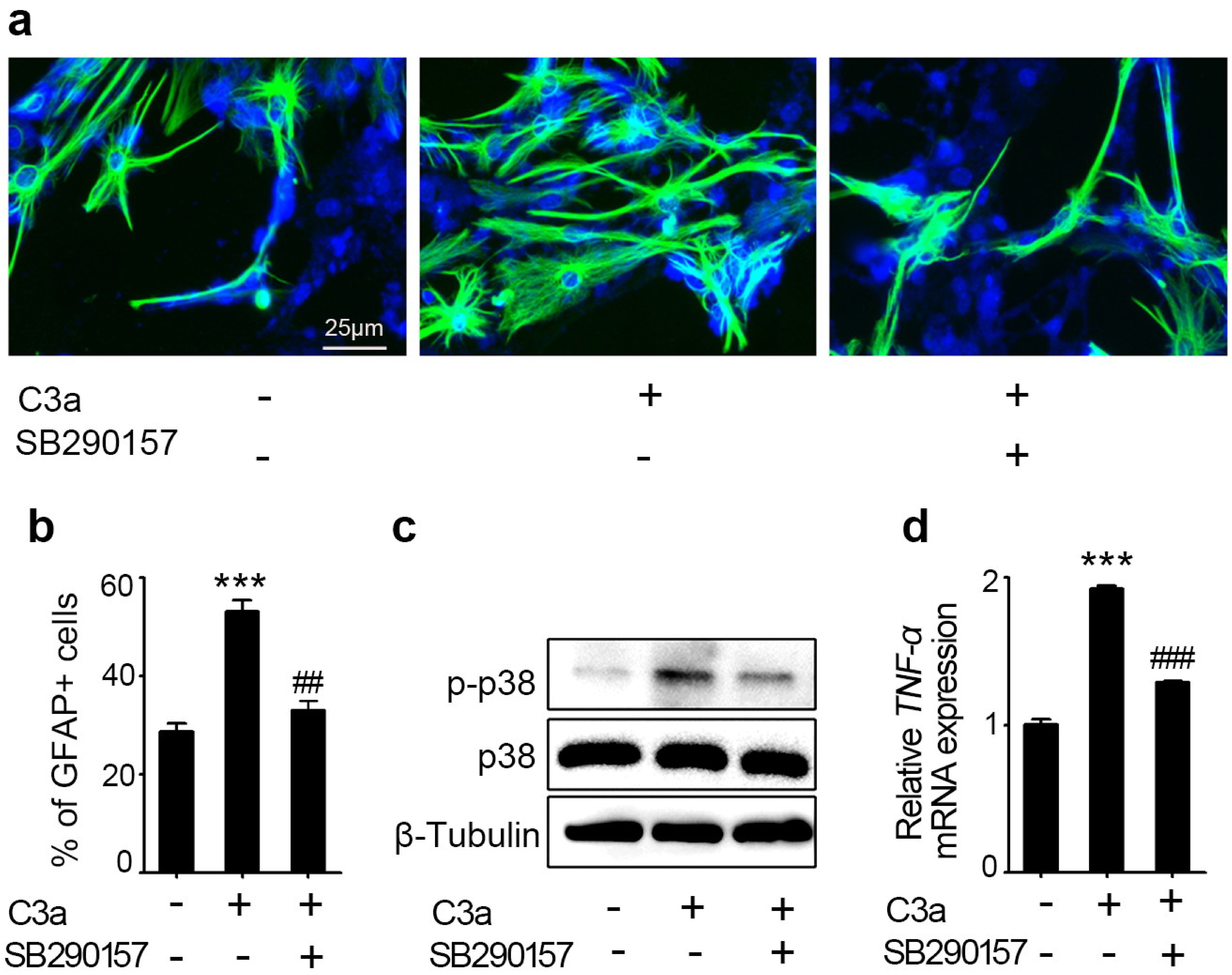

Supplementary Fig. 5 The GFAP and TNF-a expression and the phosphorylation of p38 in C3a-administrated astrocytes is inhibited by C3aR antagonist SB290157

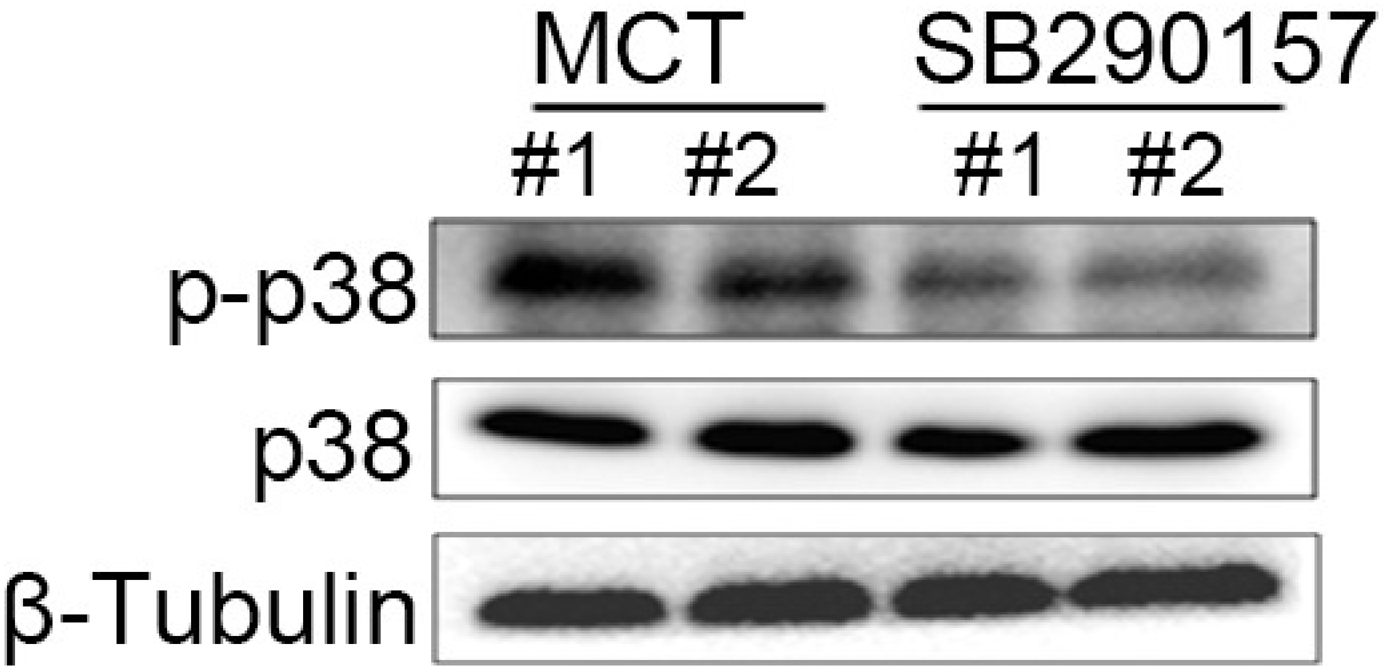

Supplementary Fig.6 C3aR antagonist treatment inhibits the p38 MAPK pathway acivation in tumor tissue of subcutaneous MB mice

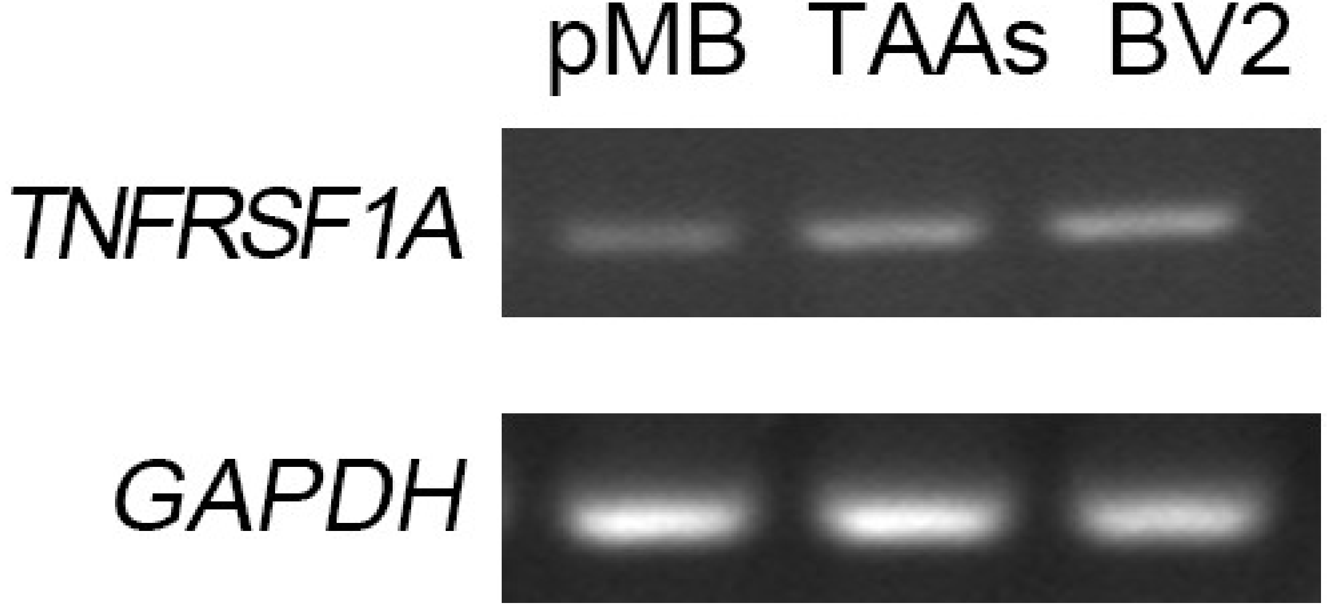

Supplementary Fig.7 *TNF-αR* is expressed in primary MB cells
